# CRISPR/Cas9 mediated high efficiency knockout of the eye color gene *Vermillion* in *Helicoverpa zea* (Boddie)

**DOI:** 10.1371/journal.pone.0197567

**Published:** 2018-05-17

**Authors:** Omaththage P. Perera, Nathan S. Little, Calvin A. Pierce

**Affiliations:** Southern Insect Management Research Unit, USDA-ARS, Stoneville, MS, United States; Oxford Brookes University, UNITED KINGDOM

## Abstract

Among various genome editing tools available for functional genomic studies, reagents based on clustered regularly interspersed palindromic repeats (CRISPR) have gained popularity due to ease and versatility. CRISPR reagents consist of ribonucleoprotein (RNP) complexes formed by combining guide RNA (gRNA) that target specific genomics regions and a CRISPR associated nuclease (Cas). The gRNA targeting specific gene sequences may be delivered as a plasmid construct that needs to be transcribed or as a synthetic RNA. The Cas nuclease can be introduced as a plasmid construct, mRNA, or purified protein. The efficiency of target editing is dependent on intrinsic factors specific to each species, the target gene sequence, and the delivery methods of CRISPR gRNA and the Cas nuclease. Although intrinsic factors affecting genome editing may not be altered in most experiments, the delivery method for CRISPR/Cas reagents can be optimized to produce the best results. In this study, the efficiency of genome editing by CRISPR/Cas system in the bollworm, *Helicoverpa zea* (Boddie), was evaluated using ribonucleoprotein (RNP) complexes assembled by binding synthetic gRNA with purified Cas9 nuclease engineered with nuclear localization signals to target the *vermillion* (eye color) gene. Mutation rates of adults emerging from embryos microinjected with 1, 2, or 4 μM RNP complexes were compared using replicated experiments. Embryos injected with 2 or 4 μM RNP complexes displayed significantly higher mutation rates (>88%) in surviving adults compared to those injected with 1 μM. The hatch rate in embryos injected with RNP complexes and with injection buffer only (mock injections) was reduced by 19.8(±5.2)% compared to noninjected control embryos, but did not differ significantly between injected embryos. Evaluation of potential off-target sites in *H*. *zea* genome did not identify any mutations. This study demonstrates that *in vitro* assembled synthetic RNP complexes can be used to obtain high genome editing rates in a reproducible manner in functional genomics or genetic manipulation studies.

## Introduction

Development of clustered regularly interspersed palindromic repeats (CRISPR) and associated RNA guided Cas nucleases has progressed rapidly since its discovery in 1987 [1–9]. CRISPR is widely used for editing genomes of various organisms ranging from bacteria to mammals. With more than 7,400 manuscripts published from 2005 to 2018, CRISPR has arguably become the most frequently used tool for *in vivo* site-directed mutagenesis (for an overwiev, see [10]). Recent reviews indicate that CRISPR-mediated genome editing has successfully been conducted in 27 arthropods, of which 23 were insects from five orders including 11 lepidopteran species (e.g. [11–18]).

In insects, genome editing has been achieved by microinjecting freshly laid eggs (embryos) with various combinations of CRISPR reagents. Production of guide RNA (gRNA) targeting the DNA sequences of interest has been carried out using plasmid constructs, *in vitro* transcription, or chemical synthesis (e.g. [19–24]). Similarly, Cas nucleases have been produced *in situ* by microinjecting plasmid constructs or mRNA, or delivered into embryos as purified protein [14,19,25–28]. Regardless of the mode or mechanism of gRNA and Cas nuclease delivery, functional ribonucleoprotein (RNP) complex formation by the two components is crucial for successful editing of DNA. In embryo microinjections, success and efficiency of heritable genome editing are dependent on a number of factors including timing of CRISPR/Cas delivery, concentration of the RNP complex, and specificity of gRNA to the target sequence. Formation of the RNP complex from the gRNA and Cas nuclease reagents prior to beginning of embryonic cell division is a critical step in achieving germline editing. Therefore, any delay in transcription of gRNA and Cas from plasmid constructs or translation of Cas mRNA may lead to reduced germline editing efficiency as well as multiple “chimeric” mutations in the embryonic cell population (e.g. [29]). In addition, RNA transcription from plasmids is highly dependent on the promoter sequence; a promoter from one species may not work optimally in a different species. Similarly, mRNA translation efficiency may differ among insects, resulting in highly variable concentrations of functional gRNA/Cas RNP complexes from identical constructs in different species. Microinjecting RNP complexes pre-assembled from synthetic gRNA and purified Cas9 proteins for genome editing is gaining popularity due to the convenience of using commercially prepared high purity reagents and the ability to deliver precise RNP concentrations that facilitate reproducibility of experiments. [30–34]. In addition, quantitative delivery of reagents increase the fidelity of comparative studies between species or between different gene targets in a single species.

Type II CRISPR-Cas systems from bacteria consist of two different RNA molecules: CRISPR RNA (crRNA) and trans-activating CRISPR RNA (tracrRNA) [35–37]. In bacteria, tracrRNA is involved in maturity of crRNA and form functional guide RNA (gRNA) by base pairing of complementary nucleotide sequences present in both molecules. Therefore, the universal tracrRNA molecule can be used to target any number of DNA sequences by simply synthesizing the crRNA molecules specific to each target. These two components can be annealed *in vitro* and bound with Cas nuclease stoichiometrically to yield RNP complexes of precise concentrations. Furthermore, performance of the Cas nuclease has been improved through the addition of nuclear localization signals (NLS) from various proteins such as SV40 large T-antigen and nucleoplasmin [38,39]. These NLS sequences are recognized by chaperones (e.g. importin α) and are actively transported to the nucleus, facilitating interactions with chromatin. The objective of this study was to identify optimal concentration of RNP complexes for high efficient genome editing in the bollworm, *Helicoverpa zea* (Boddie). We used synthetic crRNA targeting a morphological marker, the tryptophan 2,3-dioxygenase (TO) gene, which is the first enzyme in the ommochrome synthesis pathway [40]. The loss of function mutations in the TO gene in some insects (e.g. *Drosophila melanogaster* and *Tribolium castaenum*) alters wild type eye color to produce a vermillion phenotype. Guide RNA generated by annealing TO crRNA and tracrRNA was combined with a Cas9 nuclease to assemble RNP complexes for quantitative microinjection of embryos to optimize gene editing in *H*. *zea*.

## Material and methods

### Tryptophan 2, 3-dioxygenase (TO) gene sequences

The genome of an *H*. *zea* female from the SIMRU laboratory colony was sequenced and assembled by a service provider (Hudson Alpha, Huntsville, AL) using the Chromium Genome Solution platform and Supernova v1.1.5 genome assembly platform (10X Genomics, Pleasanton, CA). The draft genome was used to create a searchable database using BlastStation-Local software v1.5 (TM Software, Inc., Arcadia, CA). The cDNA sequence of *H*. *zea* tryptophan oxygenase (accession number MG976796) was used to search the database to identify a scaffold containing the tryptophan oxygenase gene. Exons of the TO gene were identified and annotated using the mRNA nucleotide sequence of *H*. *zea*. The National Center for Biotechnology Information (NCBI) peptide database was also searched using a putative *H*. *zea* TO peptide sequence translated from the cDNA sequence to identify other insect TO sequences for use in alignments and phylogenetic tree construction. Peptide sequences retrieved from the databases were aligned using the AlignX module of Vector NTI 11.5 Suite and a phylogenetic tree was generated using MEGA 6 [41].

### Guide RNA design and ribonucleoprotein complex preparation

CRISPR RNA (crRNA) used in the experiments was designed using tools available at http://crispr.mit.edu [42]. Exon sequences of the TO gene were submitted to the design pipeline. The best design for each target location was used to purchase Alt-R™ CRISPR crRNA from IDT DNA (www.idtdna.com) that could be annealed with Alt-R™ CRISPR-Cas9 tracrRNA to form a functional gRNA for each target. Alt-R™ CRISPR-Cas9 tracrRNA and Cas9 nuclease of *Streptococcus pyogenes* that was engineered to contain one N-terminal nuclear localization sequence (NLS) and two C-terminal NLSs (Alt-R™ S.p. Cas9 Nuclease 3NLS) were purchased from IDT DNA (Madison, WI). Two crRNAs for the TO gene and tracrRNA were resuspended separately in 10 mM Tris-EDTA (TE) buffer to yield a 100 μM final concentration. Each TO crRNA was combined with tracrRNA in nuclease-free tubes to yield a 10 μM final concentration in 1X duplex buffer (30 mM HEPES, pH 7.5; 100 mM potassium acetate; IDT DNA). The mixture was heated to 95°C for 5 min on a heat block and slowly cooled to room temperature to anneal the complementary nucleotide sequences in crRNA and tracrRNA to generate functional gRNA. Annealed gRNA for each target was combined with Alt-R™ S.p. Cas9 Nuclease 3NLS in 1X injection buffer (5 mM KCl; 0.1 M sodium phosphate, pH 6.8) to yield final concentrations of 1, 2, and 4 μM and incubated on ice for 15 min to facilitate the formation of ribonucleoprotein (RNP) complexes.

### Insects and eggs

A laboratory colony of *H*. *zea* maintained at the USDA-ARS Southern Insect Management Research Unit (SIMRU), Stoneville, MS was used in all experiments. The *H*. *zea* colony is routinely maintained using the methods described by Gore et al. [43]. Pupae were obtained from the colony and freshly emerged adults were placed in one gallon paper containers with 3% sugar solution for 3–4 days to facilitate mating followed by transfer of adult females to a 5 x 5 mm wire mesh cage. Wax paper was wrapped around the wire mesh cage, which was subsequently placed inside a laboratory cabinet for 30 min to collect eggs. At the end of 30 min period, the eggs were dislodged from the wax paper using a fine paint brush and mounted on approximately 1 mm wide strips of double-sided tape (3M, St. Paul, MN) attached to the 25 x 25 mm plastic coverslips. Eggs mounted on coverslips were placed in a desiccator containing silica gel and approximately -100 KPa (-30.5 inches of mercury) vacuum was applied for 10 min to dehydrate the eggs.

### Microinjections and insect rearing

Injection needles were prepared by pulling siliconized 10 μl quartz micro-capillaries using a Sutter P2000 CO_2_ laser based horizontal micropipette puller (Sutter Instruments, Novato, CA) and beveled with a BV-10 beveller (Sutter Instruments, Novato, CA). Approximately 3 μl of each injection mix (RNP complex or injection buffer) was backfilled into an injection needle. Eggs were injected using a Narishige micromanipulator model MMN-333 (Narishige International USA, Inc., Amityville, NY) and an Olympus SZ stereo microscope (Olympus Corporation, Waltham, Massachusetts) equipped with a mechanical stage. Each egg was injected with approximately 5 nl of injection mix. Mock injections (negative control) contained only the 1X injection buffer. All microinjections were completed within 30 min of egg collection so that the eggs would be no more than 60 min old at the time of injection. Control hatch rate was calculated using the eggs processed up to the desiccation stage, but not subjected to microinjection. Four replicate injections (*n* = 100 to 120 eggs) were performed with each experimental and negative control RNP complexes. Coverslips containing injected eggs were placed into 100 mm plastic petri dishes layered with a moist filter paper and covered with a lid. Petri dishes were sealed with plastic tape and placed in a plastic box designated as secondary containment. Eggs were incubated at room temperature for approximately 4 hours prior to transferring to a designated incubator which was set to 26°C and 80% RH. Eggs were observed for larval hatch and neonates were transferred to diet cups containing meridic diet [43]. Eye color of adults was recorded and any insects that had a color different from the wild type eye color were genotyped by sequencing amplicons. Hatch rates of injected eggs were corrected for control hatch rate using Henderson-Tilton's formula [44].

### Genotyping and DNA analysis

Eye color mutants, except for those used in crossing experiments, and the wild type insects from injections were frozen for genetic analysis. Genomic DNA was extracted from legs or thorax using Master Pure DNA extraction reagents as described by Perera et al. [45]. Purified DNA from each insect was re-suspended in 20 μl of 10 mM Tris-HCl. Forward and reverse primers were designed to anneal within intron 5 (3788Hz_TO_11500F: 5’-CCATTCTTCTATCTGCCGTCAT-3’) and intron 6 (3789Hz_TO_12084R: 5’-GCCCGATTCGAAAACTTCTT-3’), respectively, of the TO gene. PCR amplifications were performed on a PTC-200 DNA Engine (BioRad, Hercules, CA) with a thermal cycling profile containing 30 second initial denaturation at 95°C, followed by 35 cycles of 10 sec denaturation at 95°C, 10 sec annealing at 52°C, and 45 sec extension at 72°C. Amplification products were visualized by electrophoresis on 0.8% agarose gels (Invitrogen, Carlsbad, CA) using Tris-Acetate-EDTA (TAE) buffer (40 mM Tris-Acetate, 1mM EDTA, pH 8.0). Amplicons were cleaned by binding to AmPure XP paramagnetic beads (Beckman Coulter, Beverly, MA) at DNA:Beads (v/v) ratio of 1:1.8. Nucleotide sequences of the purified amplicons were obtained by direct sequencing with Sanger dideoxy method using the same primers as for PCR amplification (USDA-ARS Genomics and Bioinformatics Research Unit (GBRU), Stoneville, MS). Nucleotide sequence analyses were carried out using Vector NTI Advance v11.5 suite.

### Off-target sequence analysis

Seed sequence of the protospacer regions (i.e. 12 nucleotides from the 3’-end of the crRNA molecules, upstream of PAM) were used to search published *H*. *zea* genome (GCA_002150865.1) [35,46] using BlastStation-Local software v1.5 software to identify genomic scaffolds with potential off-target sites. Off-target sequences with 0–3 mismatches in the 12-nucleotide seed sequence and contained a 5’-NGG-3’ PAM sequence [35,42,47] were selected for further review. Primer pairs were developed to amplify four of the off-targets with less than 2 mismatches in the seed sequence and the lowest possible total mismatches with the target crRNA (protospacer) sequences. Genomic DNA from 48 eye color mutants resulting from the highest concentration (4μM) of RNP complex injections were amplified with the primer pairs for off-targets and the resulting amplicons were direct sequenced using the primers used for amplification. Nucleotide sequence data were analyzed with Vector NTI 11.5 Advance suite (Invitrogen, Carlsbad, CA).

### Insect husbandry

Insects were reared at 28°C, 80% RH, and 14:10 L:D cycles in environmental chambers (Percival Scientific, Perry, IA). Insects with mutations (P_0_) at the target sites were selected and mated as single pairs with moths of the opposite sex from the SIMRU *H*. *zea* laboratory colony. Resulting heterozygotes (F_1_) from each single-pair mating were inbred to obtain F_2_ progeny. Fourth instar F_2_ larvae were genotyped by sequencing the DNA extracted from a drop of hemolymph obtained by clipping the tip of one of the abdominal pseudo-legs. The number of the homozygous mutants, heterozygotes, and homozygous wild type insects were recorded for each cross.

## Results

### cDNA and genomic structure of tryptophan 2,3-dioxygenase in *H*. *zea*

**Fig 1:**
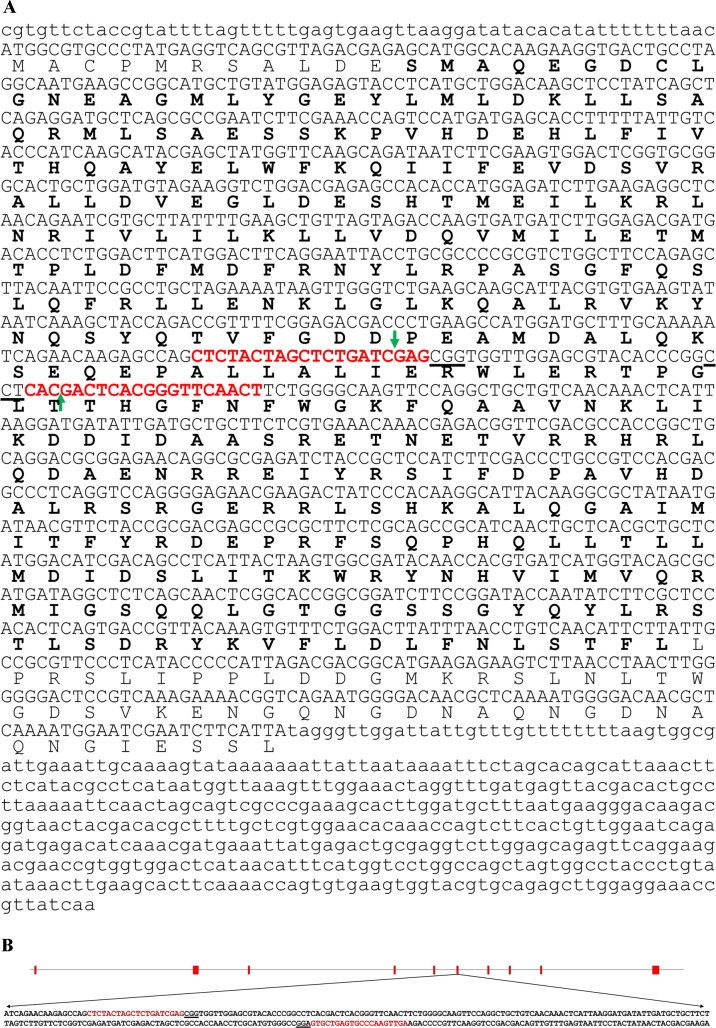
cDNA sequence, gene organization, and the CRISPR RNA (crRNA) designed for editing targets in the exon 6 of the tryptophan 2,3-dioxygenase (TO) gene in *Helicoverpa zea*. **(A)** Open reading frame of the cDNA sequence is in upper case letters and the amino acids of the tryptophan dioxygenase domain of the TO are indicated in bold text. Target sequences (protospacer) for two crRNA are shown in bold red text. Protospacer adjacent motif (PAM) and double-strand DNA break site for each target site are shown by underlined text and green arrows, respectively (accession number MG976796). (B) The TO gene consists of 10 exons (red boxes) ranging from 73 to 199 bp and introns ranging from 475 to 4323 bp in size, spanning 17,396 bp. The nucleotide sequences of crRNA targets are shown in red text and protospacer adjacent motifs (PAM) are underlined (accession numberMF598173).

Alignment of the putative amino acid sequence derived from *H*. *zea* cDNA sequence with other insect TO peptide sequences and phylogenetic analysis verified the authenticity of the *H*. *zea* TO sequence. For a more detailed description please see Supporting information (S1 and S2 Figs). cDNA sequence of *H*. *zea* TO and the translated amino acid sequence are shown in Fig 1A. A search of the *H*. *zea* genome database with the TO cDNA sequence identified a genomic DNA scaffold (Scaffold 97620) containing the full length TO gene, which was extracted, annotated, and deposited in the GenBank database under the accession number MF598173. The TO gene consisted of ten exons ranging from 73 to 199 bp in size that spanned 17,396 bp of the genome sequence (Fig 1B). The nucleotide sequence of exon 6 generated a number of high quality crRNA designs (http://crispr.mit.edu) and two of the crRNAs with the best scores were selected for editing the TO gene in *H*. *zea* (Fig 1B, red text). All crRNA designs generated are given in the Supporting information (S3 Fig).

### Egg hatch and mutation rates

Hatch rates of eggs injected with different concentrations of RNP complexes were adjusted for noninjected control hatch rates using the Henderson-Tilton formula [44] and induced mutation rates of adults emerging from control and injected eggs are shown in Table 1. The *H*. *zea* laboratory colony, which is highly inbred, produced low hatch rates ranging from 36.6 to 42.9% with an average (±stdev) of 40.2(±2.6)%. Embryo mortality due to injection, after correction for control hatch rate, was estimated to be 19.8(±5.2)%. Rates of adult emergence ranged from 87.1(±3.5)% in uninjected controls to 77.7(±15.2)% in 4.0 μM RNP injections (Table 1). Mutation recovery rates were positively correlated with the concentration of gRNA (p<0.01). Eggs injected with a standard concentration of 1 μM RNP produced significantly less mutants than the 2 and 4 μM RNP concentrations, and there was no difference between mutation rates of the two highest concentration injections (Table 1). Although a reduction of hatch rate was observed in all injected eggs, differences in hatch rates between mock injections and RNP injected eggs were not significant, indicating that RNP complexes had no deleterious effects on embryo viability.

**Table 1:** Average number pf eggs injected, average hatch count, corrected hatch count, percentage of adults emerged from larvae, and the percentage of mutants from control, mock injected (with injection buffer), and 1, 2, and 4 μM ribonucleoprotein complexes targeting exon 6 of the tryptophan 2,3-dioxygenase gene in *Helicoverpa zea*.

	Total # eggs (Avg.)	Avg. Hatch count±SD	Corrected Avg. Hatch count	% adults emerged±SD	% mutants±SD^‡^
Uninjected Control	448 (112.0)	42.3±2.6	100.0	87.1±3.5	0.0
Mock Injected	560 (116.0)	32.3±5.2	80.5	87.6±4.3	0.0
1.0 μM injected	488 (122.0)	32.2±3.3	87.2	79.1±7.5	48.1±15.1^a^
2.0 μM Injected	570 (142.5)	30.0±5.0	74.7	83.3±5.9	88.8±3.2^b^
4.0 μM Injected	541 (135.3)	35.9±5.0	78.6	77.7±15.2	93.5±6.3^b^

^‡^ Percentages of mutants from CRISPR/Cas9 injections that were significantly different are indicated by different letters.

### Mutant phenotypes and genotypes

**Fig 2:**
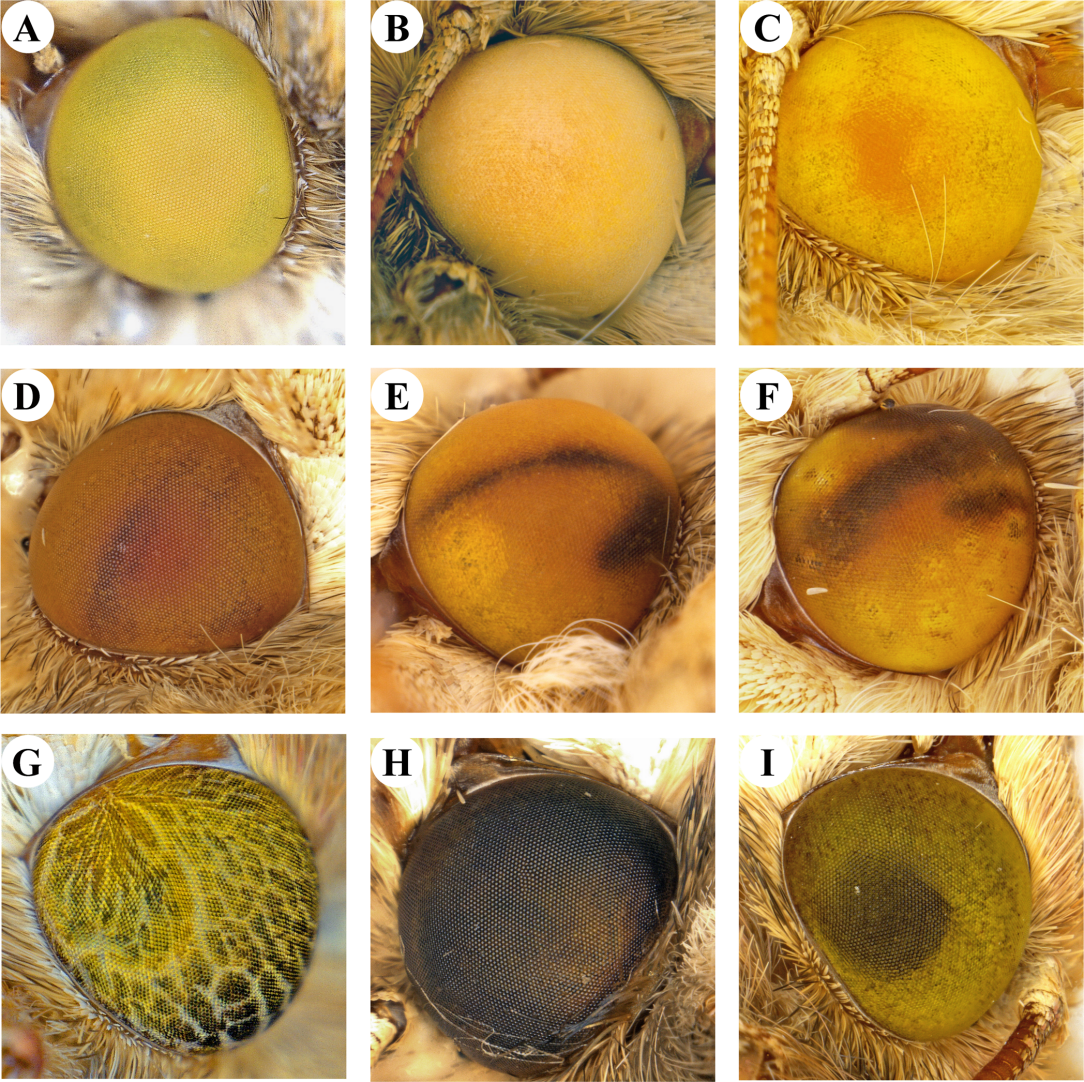
Variation in eye color in tryptophan 2,3,-dioxygenase (TO) mutants of *Helicoverpa zea*. Yellow eye phenotype of TO mutants inbred for seven generations (A) and different shades of orange color eyes observed in P_0_ (microinjected) adults (B and C). Various mosaic eye color patterns were also observed in some of the P_0_ mutants (D-G). Dark black-brown pigmentation (H) and translucent green with a dark middle (I) are common eye color phenotype variations found in the *H*. *zea* colony used in the experiments.

Loss of function TO mutants of *H*. *zea* yielded mostly yellow eye color, with only a few P_0_ insects displaying light pink to dark pink eyes (Fig 2). In some insects the pink eye color gradually turned to yellow within a few days after eclosion. Matings between P_0_ insects with the darkest pink eye color produced insects with yellow eyes in F_1_ and successive generations and no wild type eye color was observed even after seven successive generations of inbreeding.

**Fig 3:**
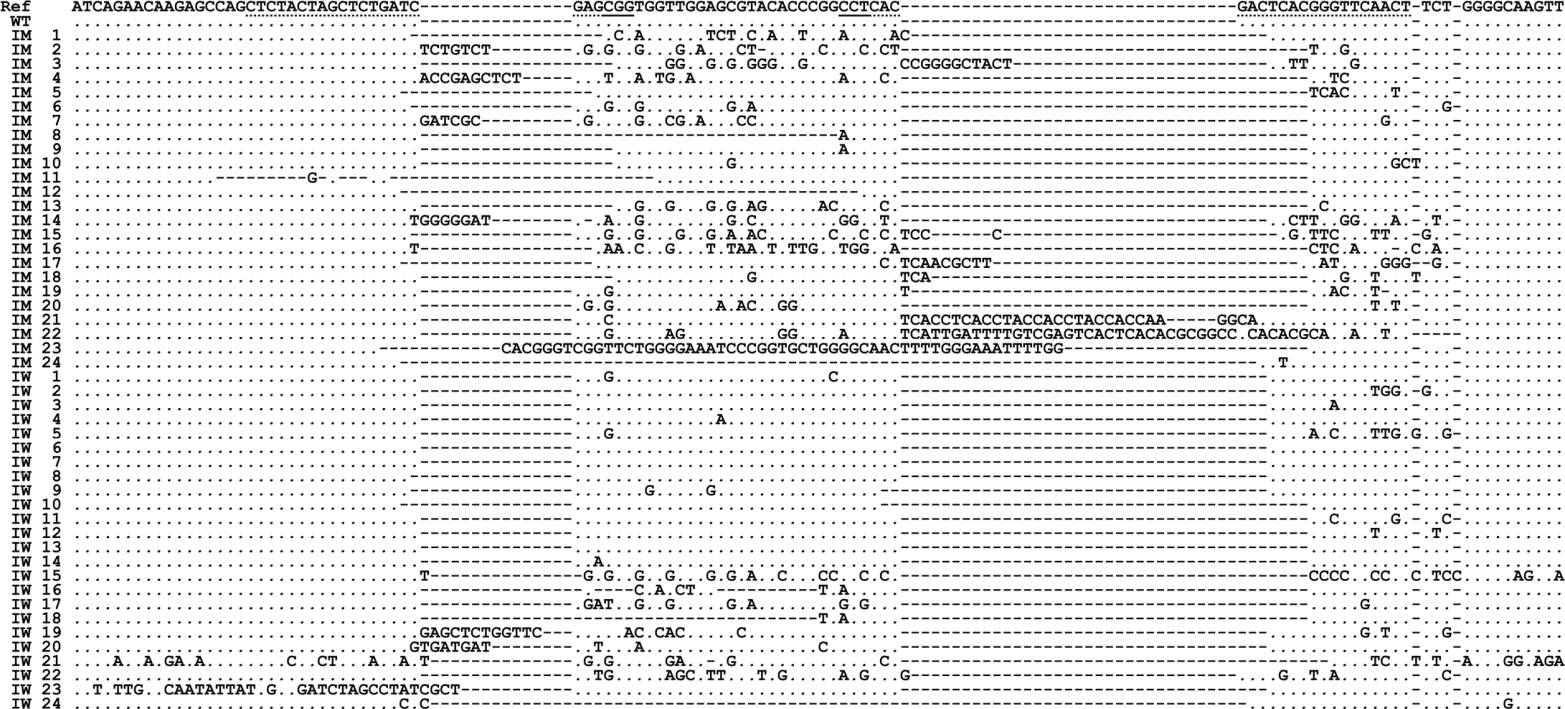
A representative sample of nucleotide sequences of the eye color mutants and wild type eye color insects from CRISPR injection experiments. Sequences IM 1 through IM 24 and IW 1 through IW 24 are from insects with mutant and wild type eye color phenotype, respectively, from CRISPR/Cas9 injections. Partial nucleotide sequences of exon 6 of the TO gene from the reference sequence (Ref) and uninjected wild type (WT) are also shown. Nucleotides identical to the exon 6 of the reference sequence (accession: MF598173) are indicated by a dot (.) and alignment gaps are shown by a hyphen (-). Nucleotides that differ from the reference are indicated on each sequence. Target (protospacer) sequences for which crRNA were designed are underlined with dotted lines and protospacer adjacent motifs (PAM) are underlined with a solid line.

The nucleotide sequence (n = 110) of the TO gene was obtained from one non-injected (WT) insect, 86 eye color mutants (including 31 insects used in mating experiments), and 24 injected insects that were classified as having wild type eye color. Alignments of sequence data with a reference identified a diverse array of mutant genotypes that included different combinations of deletions and insertions in one or both target sites (Fig 3 and S4 Fig). The number of various mutant genotypes identified in each category are summarized in Table 2. Mutations at both targets sites were identified in 89.53% of the nucleotide sequences from eye color mutants and remainder (10.47%) were mutations only at the second target site. Mutations at only the first target site were not identified in this analysis indicating a difference in efficacy between two gRNA designs. Surprisingly, all 24 insects originally classified as wild type eye color had mutations at one or both target sites (Fig 3, sequences IW 1 to IW24). Seven of these insects had one codon deletion at the second target site while another had a 12 bp insertion at the first target site in addition to the one codon deletion at the second target site. Other sequences from the phenotypically wild type insects (IW 9, IW 19, and IW 21) had insertion-deletion combinations at both target sites but preserved the open reading frame except for either insertion or deletion of a few amino acids. Careful examination of the eye colors of these insects under a microscope did not reveal any recognizable deviations from wild type eye color. Although the frequencies of yellow eye phenotypes observed in this study for 2 and 4 μM injections were between 88.8 and 93.5%, with the wild type eye color insects that had mutations actual genome editing rate may be close to 100%, (Table 1).

**Table 2:** Summary of mutation type combinations identified in tryptophan 2,3-dioxygenase gene target site nucleotide sequences of 86 yellow eye and 24 wild type insects of *Helicoverpa zea*.

Combination	Site 1	Site 2	# in Yellow Eye	# Wild Type
1	WT	Deletion	9	17
2	Deletion	WT	0	0
3	WT	Insertion	2	0
4	Insertion	WT	0	0
5	Deletion	Insertion	7	0
6	Insertion	Deletion	27	4
7^‡^	Deletion	Deletion	33	2
8	Insertion	Insertion	8	1
		Grand Total	86	24

^‡^ Includes complete deletions of the nucleotide sequences in between the target sites.

### Off-target sequence analysis

A search of *H*. *zea* genome (GCA_002150865.1) with the protospacer seed sequences identified potential off-targets in scaffolds 522 and 1430 for gRNA 1 and in 40, 180, 185, 1210, 1570, and 2020 for gRNA 2 (Supporting information S1 Table). All identified off-target sites had seven to ten total mismatches with the crRNA sequences and those with less than eight total mismatches and a maximum of two mismatches in the seed sequence regions were identified in scaffolds 185, 522, 1210, 1430, and 1570. Mutations in the Cas9 cleavage sites and the 3’-end of the protospacer region (close to PAM) inhibit Cas9 nuclease activity [35,47]. Therefore, off-target sequences with mismatches within 10 nucleotides from the 3’- end of the protospacer were not considered for analysis. Based on above criteria, primer pairs were developed for PCR amplification and nucleotide sequencing of off-target regions in genomic scaffolds 185, 1210, 1430, and 1570 (Supporting information S1 Table). Off-target sequences in scaffolds 185, 1210, and 1480 did not have any identifiable genes within 2 kbp of flanking regions. The off-target sequence of the scaffold 1570 was located in intron 1 of the transcription factor Sox-8-like gene of *H*. *armigera* (accession: XP_021195044.1). Genomic DNA sequences amplified using primers to scaffold 1210 were polymorphic in some insects and were not likely to be off-targets (S5B Fig). Therefore, only some of the insects had off-target sequences matching scaffold 1210 (S5B Fig). Both wild type and yellow eye mutants had significant differences in the off-target sequence compared to scaffold 185 from the published genome sequence (S5B Fig). This is most likely due to genome level differences between insect colonies used for genome sequencing and the TO gene editing experiments. Evaluation of all nucleotide sequences from the selected off-target regions in yellow eye mutants revealed no evidence of non-specific activity, indicating that both gRNA used for mutating the TO gene was specific to the target regions (Supporting information S5 Fig, panels A-D).

## Discussion and conclusions

In this study, the efficacy of in vitro assembled riboprotein complexes containing synthetic tracrRNA, crRNA, and a purified Cas9 nuclease in generating mutations in *H*. *zea* was studied using different RNP concentrations to target the *H*. *zea* TO gene. The microinjection process reduced egg hatch by 19.8%, but there was no significant difference in egg hatching between injections of control solutions or ribonucleoprotein complexes targeting the TO gene. Mutation rates, however, were much higher when injected with 2 and 4 μM concentrations of TO ribonucleoprotein complex compared to eggs injected with the manufacturer recommended 1 μM concentration. In addition, the mutation rates at 2 and 4 μM RNP concentrations were much higher than those obtained by using different combinations of gRNA and Cas9 nuclease delivery methods in other lepidopteran species (e.g [23,24,48,49]). Therefore, we conclude that 2 μM or higher concentrations of ribonucleoprotein complex were efficient in generating desired mutations in *H*. *zea*. However, it may be necessary to optimize the RNP complex concentrations in experiments with other species or when using gRNA for different targets to achieve the best results. Furthermore, it was observed that greater than 6 μM concentrations of ribonucleoprotein complexes tend to clog the injection needles quickly during injections, reducing reproducibility and rendering the process much more difficult.

Mutation rates observed in this study with 2 μM or higher RNP concentrations were greater than most of the reported mutation rates produced by injecting various combinations of cDNA, mRNA, and purified Cas9 protein. Injection of DNA constructs (plasmids) coding for gRNA and Cas nuclease has typically produced low heritable mutation rates [19,50] compared to studies that employed transcribed gRNA with Cas protein or mRNA. For example, Markert et al [23] used transcribed gRNA and Cas9 mRNA in the monarch butterfly, *Danaus plexippus*, and reported that injections of two gRNAs complementary to exons two and three of the c*ry2* gene at 0.1 μg/μl together with 0.5 μg/μl of mRNA encoding Cas9 resulted in 21.6% larval hatch rate, 61.5% somatic mutation rate, and 100% germline mutation rates in the progeny of mutants tested by crossing (n = 2). However, the exact percentage of germline mutations is not clear because all mutants were not crossed to obtain progeny for testing and the experiments were not replicated. In another study, Li et al. [48] used transcribed gRNA and mRNA for Cas9 at various concentrations to knock out *Abdominal-B*, *ebony*, and *frizzled* genes in *Papilio xuthus* to achieve morphological mutation rates from 18.3 to 90.9%, in a locus and RNA concentration dependent manner. In most experiments, higher concentrations of dual gRNA and Cas9 mRNA produced higher rates of mutations in *P*. *xuthus*. In a similar study on *Bombyx mori wnt1* mutations with transcribed gRNA and mRNA for Cas9, high rates of mutations were observed in experiments that used high concentrations of Cas9mRNA and gRNA (300 ng/μl) compared to low concentrations (30 ng/μl) of gRNA [51]. However, egg hatch percentage was 6.6% in 300 ng/μl gRNA injections compared to 28.8% in 30 ng/μl gRNA injections. Again, the experiments were not replicated and statistical analyses were not provided. Varying rates of mutations produced using injections of Cas9 protein and transcribed gRNA have also been reported in the butterfly species *Junonia coenia* and *Vanessa cardui* [24] and a species of moth, *Spodoptera littoralis* [52]. Mutating *spalt* and *distal-less* genes in *J*. *coenia* and *V*. *cardui* using an injection mixture containing 100 ng/μl gRNAs and 200 ng/μl Cas9 protein yielded mutation rates 33.3and 56.0% for *spalt* and 41.0 and 51.7% for *distal-less*, respectively. Although *J*. *coenia* mutation rates were lower than that of *V*. *cardui* in both experiments, it is not known whether high mutation rates could be achieved by using different concentrations of reagents because only one combination of gRNA and Cas9 protein concentrations was reported. In a study targeting half-ATP binding cassette (ABC) transporters in *Helicoverpa armigera*, Khan et. al. [53] used *in vitro* transcribed mRNA and sgRNA at 200 and 25 ng/μl concentrations, respectively, to successfully mutate the target genes. However, data pertaining to the efficiency of gene knockouts were not reported. It should be noted that although comparisons between different reagent combinations targeting different genes in a number of insect species showed high levels of variation in mutation efficiency (reviewed by [14]), we have not evaluated the efficacy of different reagent systems (or cannot locate any published research) on a single gene target on the same insect species. Reviews of the literature indicate that depending on the target site, similar gRNA/Cas reagent combinations could produce highly variable mutation rates within a single species (e.g. [26,48]). However, all results reported so far have been obtained by a single set of injections (i.e. no replicates) and the mutation efficiencies were not analyzed statistically. Therefore, having a precisely quantifiable reagent system such as the RNP complexes used in this study may be necessary to obtain reproducible results for comparison of mutation efficiencies between targets or identical targets in different species using replicated experiments. In addition, true comparisons among the efficiencies of different experiments were not possible due to inability to convert of the injected reagent concentrations reported in ng/μl to the concentrations of actual RNP complexes due to unknown efficiency of transcription and translation of plasmids or translation of mRNA in different species.

Loss of function mutations in the TO gene in some insect species of Coleoptera and Diptera leads to vermillion eye color [54–56]. RNA interference (RNAi) of the TO gene indicated knock down of eye pigment intensity in *Plodia interpunctella* larvae, but no definitive color was reported, most likely due to inability to differentiate colors of eye spots in larvae [57]. While most *H*. *zea* TO mutants had yellow eye color, others ranged from pink to light red immediately after eclosion, but in some insects, turned yellow within a few days (Fig 2). Knockout of *scarlet* that dimerizes with *white* to from the ommochrome precursor transporter in the closely related species *Helicoverpa armigera* [53] also produced yellow eye color phenotype, which was attributed to the presence of ekapterin, an eye pigment originally described in *Ephestia kuehniella* [58]. However, pink or light red eye colors observed in TO knockouts of *H*. *zea* were not detected in *scarlet* knockouts of *H*. *armigera*. Furthermore, single-pair matings between *H*. *zea* TO mutants with reddish eye color produced progeny with yellow eye color only and we postulate that the variations in eye color observed in *H*. *zea* were most likely due to differences in expression of genes involved in red and yellow eye pigment production pathways in different insects.

Pteridine and ommochrome eye pigment synthesis pathways include multiple steps in which mutations could result in different eye colors. Various proportions of different eye pigments could produce a range of eye color phenotypes. Mutations in the TO gene effectively terminated the ommochrome pigment xanthommmatin (brown) synthesis leading to unmasking of pigments produced in the pteridine pathway [40,59]. *Vermilion* eyes are produced in insects that predominantly contain the red pigment drosopterin. Although pteridines may not contribute to eye color in *Bombyx mori* [60,61], there may be pteridine pigments present in the ommatidia of other lepidopteran species including *H*. *armigera* and *H*. *zea*. Presence of a red pigment that contributed to pink hues in *H*. *zea* may indicate that there are differences in eye color pigment repertoire even in closely related species.

It is possible that wild type eye color in the insects with in-frame mutation events resulted from the unaltered functionality of the TO in mutants with these deletions or insertions. However, lack of detectable eye color change in P_0_ adults with frame-shift mutations cannot be explained except by assuming that they were chimeras in which RNP complexes were injected into embryos that already had started cell division. In such an event, cells that were not mutated could produce a functional TO enzyme to yield ommochrome pigments. This the most likely scenario in the insects with wild type eye color which had mutations that caused frame shifts that led to truncation of the enzyme. In fact, insect embryonic development begins with a syncytial stage where multiple nuclei are present within the embryo with partially formed cells [18]. In a closely related species *H*. *armigera*, a ring of syncytial nuclei cold be seen at 4 hours after oviposition [62] and nuclei division could have started much earlier than 4 hours. Observation of mosaic eye pigmentation patterns that contained patches of wild type eye color in mutants (Fig 2) and identification of mutations that abolish the TO enzyme activity in phenotypically wild type insects (Fig 3) of *H*. *zea* in this study clearly indicate the presence multiple nuclei even in embryos injected within 60 minutes of oviposition. The presence of two or more nuclei at the time of injection may be attributed either to start of syncytial nuclei division in *H*. *zea* embryos before 60 min or to females holding the fertilized eggs for some time before oviposition. Although a scientifically intriguing matter, this subject was out of the scope of this research and was not further investigated.

Mutations in off-target genomic regions have the potential to generate unexpected complications in genome editing experiments [63]. Undesirable mutations can be significantly reduced by designing high quality gRNA with minimal off-target effects or high fidelity Cas9 nucleases requiring specific target binding by gRNA [64–66]. Although optimal crRNA designs were used in this study, the Cas9 nuclease utilized was not a high fidelity enzyme. Scanning of the *H*. *zea* genome for off-target sequences identified several potential sites. However, PCR amplification and nucleotide sequencing of four putative off-target sequence regions in mutant yellow eye insects did not identify any unintended mutations (Supporting information S5 Fig, panels A-D). Therefore, we can confidently state that crRNA designs used in this study were highly specific to the intended targets and mutant phenotypes observed were a direct result of mutations in the TO gene.

In conclusion, this study demonstrates the utility of in vitro assembled RNP complexes made of synthetic guide RNA components and purified Cas9 nuclease containing NLS in a series of replicated experiments using known concentrations of RNP complexes. In *H*. *zea*, 2 μM or higher concentrations of RNP complexes targeting the TO gene produced greater than 90% mutants in adults the developed from injected eggs. Results showed that RNP complexes can be introduced to embryos in a quantitative manner to maximize mutation rates in genome editing experiments. Efficiencies in mutating different targets within same species or an identical target between different species could be easily compared by injecting precise concentrations of RNP complexes. In addition, the ability to use precise concentrations of RNP complexes increase the reliability and precision of experimental reproducibility. This research should provide guidelines for standardizing the CRISPR/Cas mediated genome editing in non-model insects.

## Supporting information

S1 Fig Alignment of tryptophan 2,3-dioxygenase (TO) polypeptide sequences from various insect species. TO polypeptide sequences from *Heliothis virescens* (PCG74852.1), *Spodoptera litura* (XP_022823393.1), *Plodia interpunctella* (AAR24625.1), *Bicyclus anynana* (XP_023952864.1), *Papilio polytes* (XP_013141355.1), *Bombyx mori* (XP_004922659.1), *Anopheles darlingi* (ETN63322.1), *Anopheles gambiae* (XP_312204.2), *Aedes aegypti* (AAL37360.1), *Neodiprion lecontei* (XP_015514842.1), *and Zootermopsis nevadensis* (XP_021924784.1) were downloaded from National Center for Biotechnology Information and aligned using AlignX module of Vector NTI Advance 11.5 suite (Invitrogen). Identical amino acids are indicated by red text with yellow background. A block of similar amino acids are shown in black text with green background, conservative amino acids are shown in dark blue text, and non-similar amino acids are shown in black text. Alignment gaps are indicated by a hyphen (-). Tryptophan dioxygenase domain is underlined.
(PDF)

S2 Fig The evolutionary tree generated using tryptophan 2,3-dioxygenase (TO) polypeptide sequences from various insect species. A maximum likelihood evolutionary tree was constructed using TO sequences from *H*. *zea* (MG976796), *Heliothis virescens* (PCG74852.1), *Spodoptera litura* (XP_022823393.1), *Plodia interpunctella* (AAR24625.1), *Bicyclus anynana* (XP_023952864.1), *Papilio polytes* (XP_013141355.1), *Bombyx mori* (XP_004922659.1), *Anopheles darlingi* (ETN63322.1), *Anopheles gambiae* (XP_312204.2), *Aedes aegypti* (AAL37360.1), *Neodiprion lecontei* (XP_015514842.1), *and Zootermopsis nevadensis* (XP_021924784.1). Using the MEGA 6.0 we identified the Jones-Taylor-Thornton model with a discrete gamma distribution and some evolutionarily invariable sites (JTT+G+I) as the best model for gene phylogeny reconstruction. JTT+G+I model was run with 10,000 bootstrap replicates was used to generate the evolutionary tree. Lepidopteran and dipteran TO sequences were grouped in two distinct evolutionary branches with 100% bootstrap support. Hymenoptera (*N*. *lecontei*) and isopteran (*Z*. *nevadensis*) TO sequences were basal to the evolutionary tree with weak bootstrap support.
(PDF)

S3 Fig CRISPR RNA (crRNA) designs for *Helicoverpa zea* tryptophan 2,3-dioxygenase (TO) gene exon 6 generated by tools at crispr.mit.edu. Nucleotide sequences of the crRNA used in the experiments are shown in bold red text followed by protospacer adjacent sequence (PAM) in bold black text.
(PDF)

S4 Fig Alignment of the partial nucleotide sequences of the exon 6 of the TO gene from reference sequence (Ref), uninjected wild type (WT), eye color mutants from CRISPR/Cas9 injections (IM). Nucleotides identical to the exon 6 of the reference sequence (accession: MF598173) are indicated by a dot (.) and alignment gaps are shown by a hyphen (-). Nucleotide that differ from the reference are indicated on each sequence. CRISPR RNA (crRNA) target sequences and protospacer adjascent motifs (PAM) are underlined with dotted lines and solid lines, respectively.
(PDF)

S5 Fig Alignments of nucleotide sequences of the off-target sites in *H*. *zea* genomic scaffolds 185, 1210, 1430, and 1570 from yellow eye mutants. One hundred nucleotides from each scaffold, including 38–40 flanking the off-target sequence obtained from wild type insects (WT) and yellow eye (IM) mutants were aligned with *H*. *zea* DNA sequence from corresponding scaffold. Off-target sequence is marked in red text and the PAM sequence is underlined. Nucleotides identical to wild type sequence are indicated by a dot (.) and alignment gaps are indicated by a hyphen (-). Nucleotides that are different from the genomic scaffold are shown on each sequence. (A) Alignment of nucleotide sequences corresponding to the off-target site in the scaffold 185 (S185). Nucleotide sequence of the off-target site from the published genome (S185) was significantly different from those obtained from wild type and yellow eye mutants. (B) Alignment of nucleotide sequences corresponding to the off-target site in the scaffold 1210 (S1210). Two different nucleotide variants, one identical to the genomic scaffold 1210 sequence (S2010a) and the other with a number of polymorphisms (S2010b), were identified in the yellow eye mutants. Alignment of S2010a and S1210b (a) indicates that S2010b variants have additional mutations in the protospacer seed sequence and significantly different from the crRNA design for TO. Alignment of the nucleotide sequences similar to S2010a off-target region from wild type and yellow eye mutants (b) indicates that there were no off-target mutations in the insects with edited TO gene. (C) Alignment of nucleotide sequences corresponding to the off-target site in *H*. *zea* genomic scaffold 1430 (S1430). Nucleotide sequences from yellow eye mutants indicate that there were no off-target effects in the edited TO gene. (D) Alignment of nucleotide sequences corresponding to the off-target site in *H*. *zea* genomic scaffold 1570 (S1570). Nucleotide sequences from yellow eye mutants indicate that there were no off-target effects in the edited TO gene.
(PDF)

S1 Table Potential off-targets in the genome of *Helicoverpa zea*. Seed sequence (12 nucleotides upstream of the protospacer adjacent motif) of the target sequences of the TO gene was used to search genome of *H*. *zea* to identify potential off-targets that had three or less mismatches with the seed sequences of the targets and ten or less total mismatches. Primer pairs designed to PCR amplify and sequence off-target sequences are shown next to the selected sequences. Seed sequence region is shown in blue text and nucleotide mismatches are shown in lowercase text. PAM sequences are shown in bold black text. A lower case “c” at the end of the start position indicates an off-target sequence on the complementary strand of a scaffold. N/A: primers were not designed to these off-targets.
(PDF)
