# Supplementary material for: CRISPR/Cas9 mediated high efficiency knockout of the eye color gene Vermillion in Helicoverpa zea (Boddie)
Source: PLoS One. 2018 May 17;13(5):e0197567. doi: 10.1371/journal.pone.0197567 (PMC5957398; doi:10.1371/journal.pone.0197567)
Supplement: S1 Fig — TO polypeptide sequences from Heliothis virescens (PCG74852.1), Spodoptera litura (XP_022823393.1), Plodia interpunctella (AAR24625.1), Bicyclus anynana (XP_023952864.1), Papilio polytes (XP_013141355.1), Bombyx mori (XP_004922659.1), Anopheles darlingi (ETN63322.1), Anopheles gambiae (XP_312204.2), Aedes aegypti (AAL37360.1), Neodiprion lecontei (XP_015514842.1), and Zootermopsis nevadensis (XP_021924784.1) were downloaded from National Center for Biotechnology Information and aligned using AlignX module of Vector NTI Advance 11.5 suite (Invitrogen). Identical amino acids are indicated by red text with yellow background. A block of similar amino acids are shown in black text with green background, conservative amino acids are shown in dark blue text, and non-similar amino acids are shown in black text. Alignment gaps are indicated by a hyphen (-). Tryptophan dioxygenase domain is underlined. (PDF) [file pone.0197567.s001.pdf]

S1 Fig. Alignment of tryptophan 2,3-dioxygenase (TO) polypeptide sequences from various insect species. TO polypeptide sequences from *Heliothis virescens* (PCG74852.1), *Spodoptera litura* (XP_022823393.1), *Plodia interpunctella* (AAR24625.1), *Bicyclus anynana* (XP_023952864.1), *Papilio polytes* (XP_013141355.1), *Bombyx mori* (XP_004922659.1), *Anopheles darlingi* (ETN63322.1), *Anopheles gambiae* (XP_312204.2), *Aedes aegypti* (AAL37360.1), *Neodiprion lecontei* (XP_015514842.1), *and Zootermopsis nevadensis* (XP_021924784.1) were downloaded from National Center for Biotechnology Information and aligned using AlignX module of Vector NTI Advance 11.5 suite (Invitrogen). Identical amino acids are indicated by red text with yellow background. A block of similar amino acids are shown in black text with green background, conservative amino acids are shown in dark blue text, and non-similar amino acids are shown in black text. Alignment gaps are indicated by a hyphen (-). Tryptophan dioxygenase domain is underlined.

```
                           1                                                                             80
H. zea              (1)  -MACPMRSALDESMAQEGDCLGNEAGMLYGEYLMLDKLLSAQRMLSAESSKP-VHDEHLFIVTHQAYELWFKQIIFEVDS
S. litura           (1)  -MACPMRSMMDESSAQEGNHLGNEAGMLYGEYLMLDKLLSAQRMLSSESSKP-VHDEHLFIITHQAYELWFKQIIFEVDS
B. mori             (1)  -MACPMRSAIDEINGQEGAHLGNEAGMLYGEYLMLDKLLSAQRMLSAESSKP-VHDEHLFIITHQAYELWFKQIIFEVDS
H. virescens        (1)  -MACPMRSVMDDSTAQEGNHLGNEAGMLYGEYLMLDKLLSAQRMLSAESSKP-VHDEHLFIVTHQAYELWFKQIIFEVDS
P. interpunctella   (1)  -MACPMRSMNDSS--QDGSCLGNEAGMLYGEYLMLDKLLAAQRMLSAESSRP-AHDEHLFIVTHQAYELWFKQIIYEVDS
P. polytes          (1)  -MACPMRSVMDDSGAQDGSHLGNEAGMLYGEYLMLEKLLTSQRMLSAESSKP-VHDEHLFIVTHQAYELWFKQIIFEVDS
B. anynana          (1)  -MACPMRSVTDDS-AQDGDHLGNEAGMLYGEYLMLDKLLSAQRMLSAESSKP-VHDEHLFIVTHQAYELWFKQIIFEVDS
A. aegypti          (1)  -MSCPVGNHNGDP--QGGQRLGSEAGMLYGEYLMLDKVLSAQRMLSVESNKP-VHDEHLFIVTHQAYELWFKQIIFELDS
A. darlingi         (1)  -MSCPVRSGFVDS-VQGGHHLGSEAGMLYGEYLMLDKVLSAQRMLSVEGKKP-VHDEHLFIVTHQAYELWFKQIIFELDS
A. gambiae          (1)  -MSCPMRSGFVDS-VQGGHHLGSEAGMLYGEYLMLDKVLSAQRMLSVEGKKP-VHDEHLFIVTHQAYELWFKQIIFELDS
Z. navadensis       (1)  MNSCVFTNGTDIS--QEEDQLDGQAGMLYGEYLQLDKILNAQRMLSARTNHP-VHDEHLFIITHQAYELWFKQILFELDS
N. lecontei         (1)  -MACPMRNGGGEDTSQDGDQLTEGPGMLYGEYLRLDKILSAQRMLSSQCGEEGVHDEHLFIVTHQAYELWFKQIIFELDS

                          81                                                                            160
H. zea             (79)  VRALLDVE--GLDESHTMEILKRLNRIVLILKLLVDQVMILETMTPLDFMDFRNYLRPASGFQSLQFRLLENKLGLKQAL
S. litura          (79)  VRALLDVE—GLDESHTMEILKRLNRVVLILKLLVDQVMILETMTPLDFMDFRNYLRPASGFQSLQFRLLENKLGLKQAL
B. mori            (79)  VRALLNVE--GLDESHTMEILKRLNRIVLILKLLVDQVMILETMTPLDFMDFRHYLRPASGFQSLQFRLLENKLGLKQAL
H. virescens       (79)  VRALLDVE--GLDESHTMEILKRLNRVVLILKLLVDQVMILETMTPLDFMDFRNYLRPASGFQSLQFRLLENKLGLKQAL
P. interpunctella  (77)  VRSLLDVE--GLDESHTMEILKRLNRIVLILKLLVDQVMILETMTPLDFMDFRNYLRPASGFQSLQFRLLENKLGLKQAL
P. polytes         (79)  VRSLLDVE--GLDEGHTMEILKRLNRVVLILKLLVDQVMILETMTPLDFMDFRHYLRPASGFQSLQFRLLENKLGLKQAL
B. anynana         (78)  VRTLLDVE--GLDESHTMEILKRLNRVVLILKLLVDQVMILETMTPLDFMDFRHYLRPASGFQSLQFRLLENKLGLKQAL
A. aegypti         (77)  IRSLFSTE--HMEESRTLEILKRLNRIVMILKLLVDQVPILETMTPLDFMDFRDFLSPASGFQSLQFRLLENKLGVKTEH
A. darlingi        (78)  IRVLFSTE--HIEESRTLEILKRLNRIVMILKLLVDQVPILETMTPLDFMDFRDYLSPASGFQSLQFRLLENKLGVKSEH
A. gambiae         (78)  IRDLFSTE--HIEESRTLEILKRLNRIVMILKLLVDQVPILETMTPLDFMDFRDYLSPASGFQSLQFRLLENKLGVKSEH
Z. navadensis      (78)  VRAKFNTEDLVLDESQSQEILKRMNRIVLILKLLVDQVMILETMTPLDFMDFRNYLSPASGFQSLQFRLLENKLGVKQEH
N. lecontei        (80)  VRALFNTE--GLDESRTLEILKRLNRIVLILKLLVDQVMILETMTPLDFMAFRDYLCPASGFQSLQFRLLENKLGLLQEH

                         161                                                                            240
H. zea            (157)  RVKYNQSYQTVFGDDPEAMDALQKSEQEPALLALIERWLERTPGLTTHGFNFWGKFQAAVNKLIKDDIDAASRETNETVR
S. litura         (157)  RVKYNQSYQTVFGDDPEAMEALHKSEQEPALLALIERWLERTPGLNTHGFNFWGKFQAAVSKLIKDDIDAASHETNETVR
B. mori           (157)  RVKYNQNYQTVFGDDPEAMDSLQKSEQEPALLALIERWLERTPGLNTHGFNFWGKFQATVNKMLKEDIETANLESIDAVR
H. virescens      (157)  RVKYNQSYQNVFGDDPEAMDALQKSEQEPALLGLIERWLERTPGLTTRGFNFWGKFQAAVTKLIKDDIDAANETNETVR
P. interpunctella (155)  RVKYNQNYQTVFGDDPAAMDALHKSEEEPALLALIERWLERTPGLQTHGFNFWGKFQAAVNKTITDDIEAAMTEPNETVR
P. polytes        (157)  RVKYNQNYQTVFGDDPEAMESLRKSEEEPALLALIERWLERTPGLNTHGFNFWGKFQNAVDTMIKDDIDEAMLEPNEVVR
B. anynana        (156)  RVKYNQNYQTVFGDDPEAMDSLHKSEEEPALLALIERWLERTPGLITHGFDFWGKFQAAVDKMIRESVEEAMREPNETVR
A. aegypti        (155)  RVKYNQKYSEVFASDPCAIERLSITESEPSLADLVQKWLERTPGLETNGFNFWGKFEESVEQLLADQEASAMEEEHENVK
A. darlingi       (156)  RVKYNQKYTEVFASDPSAIDRIAVTEEEPSLADLVQKWLERTPGLEEDGFNFWGKFQESVEKLLSDQEASAMKEEHEHVR
A. gambiae        (156)  RVKYNQKYTEVFASDPGAIERIGTTETEPSLADLVQKWLERTPGLEQDGFNFWGKFQESVEQLLAEQEASAMSEEHENVR
Z. navadensis     (158)  RVKYNQKYSCVFGNDPEAIKAIEKSENEPPLAHLVQRWLERTPGLEADGFNFWGKYKQAVETLLAKQRKTAEAEVNETLR
N. lecontei       (158)  RVKYNQSYTRVFGRDPEAMTALGLAENEPSLSCLVQRWLERTPGLEPHNFDFWGKYTRAVEGMLTEQEASAKAEKSEQVR

                         241                                                                            320
H. zea            (237)  RHRLQDAENRREIYRSIFDPAVHDALRSRGERRLSHKALQGAIMITFYRDEPRFSQPHQLLTLLMDIDSLITKWRYNHVI
S. litura         (237)  RHRLQDAENRREIYRSIFDPAVHDALRSRGERRLSHKALQGAIMITFYRDEPRFSQPHQLLTLLMDIDSLITKWRYNHVI
B. mori           (237)  RHRLQDVENRREIYRSIFDPTVHDALRSRGERRLSHRALQGAIMITFYRDEPRFSQPHQLLTLLMDIDSLITKWRYNHVI
H. virescens      (237)  RHRLQDAENRREIYRSIFDPAVHDALRSRGERRLSHKALQGAIMITFYRDEPRFSQPHQLLTLLMDIDSLITKWRYNHVI
P. interpunctella (235)  RHRLQDAENRREIYRSIFDPAVHDALRSRGERRLSHRALQGAIMITIYRDEPRFSQPHQLLTLLMDIDSLITKWRYNHVI
P. polytes        (237)  KHRLQDAENRREIYRSIFDPSVHDALRSRGERRLSHRALQGAIMITFYRDEPRFSQPHQLLTLLMDIDSLITKWRYNHVI
B. anynana        (236)  RHRLQDAENRREIYRSIFDPAVHDALRSRGERRLSHRALQGAIMITFYRDEPRFSQPHQLLTLLMDMDSLITKWRYNHVI
A. aegypti        (235)  NYRLMDIEKRREVYKSIFDASVHDALVARGDRRFTHRALQGAIMITFYRDEPRFSQPHRLLMLLMDIDSLITKWRYNHVI
A. darlingi       (236)  EYRLMDIGKRREVYKSIFDAEVHDALVARGERRFTHKALQGAIMITFYRDEPRFSQPHQLLMLLMDIDSLITKWRYNHVI
A. gambiae        (236)  EYRLMDIDKRREVYKSIFDAQVHDALVARGERRFTHKALQGAIMITFYRDEPRFSQPHQLLMLLMDIDSLITKWRYNHVI
Z. navadensis     (238)  QHRFMDYEKRKEIFNSIFDPTIHAALVSRGERRFSHRAMQGAIMITFYRDEPRFNQPHQLLTLLMDIDSLITKWRYNHVI
N. lecontei       (238)  NHRLSDISSRRAVFETIFNEALHDALVSRGERRFTHAALQGAVMITLYRDEPRFSQPHQILTLLMDIDSLITKWRYNHVI
```

```
                           321                                                                              400
H. zea               (317) MVQRMIGSQQLGTGGSSGYQYLRSTLSDRYKVFLDLFNLSTFLLPRSLIPPLDDGMKRSLNLTWGDSVK----ENGQNGD
S. litura            (317) MVQRMIGSQQLGTGGSSGYQYLRSTLSDRYKVFLDLFNLSTFLLPRSLIPPLDDNMKKSLTLTWGDKENG---QNMQNGA
B. mori              (317) MVQRMIGSQQLGTGGSSGYQYLRSTLSDRYKVFLDLFNLSTFLLPRALIPPLDEDMKRSLNLTWGDNVR-------ENGD
H. virescens         (317) MVQRMIGSQQLGTGGSSGYQYLRSTLSDRYKVFLDLFNLSTFLLPRSLIPPLDDDMKRSLNLTWGDTVK----ENGQNGD
P. interpunctella    (315) MVQRMIGSQQLGTGGSSGYQYLRSTLSDRYKVFLDLFNLSTFLLPRSLIPPLDDGMKKSLNLTWGDNAR-------ENGK
P. polytes           (317) MVQRMIGSQQLGTGGSSGYQYLRSTLSDRYKVFLDLFNLSTFLLPRSLIPPLDDGMKRSLNLTWGDNIK----ENSQNGQ
B. anynana           (316) MVQRMIGSQQLGTGGSSGYQYLRSTLSDRYKVFLDLFNLSTFLLPRSLIPPLDDGIKRDLSLTWGDHIRENGHSAKENGE
A. aegypti           (315) MVQRMIGSQQLGTGGSSGYQYLRSTLSDRYKVFIDLFNLSTFLIPRGSIPPLTCEMQKALNLAWGSPVH----------Q
A. darlingi          (316) MVQRMIGSQQLGTGGSSGYQYLRSTLSDRYKVFLDLFNLSTFLIPRNTIPPLTNEMQKALNMAWGSPAH----------T
A. gambiae           (316) MVQRMIGSQQLGTGGSSGYQYLRSTLSDRYKVFLDLFNLSTFLIPRQSIPPLTNEMQKALNLAWGSPAH----------F
Z. navadensis        (318) MVQRMIGSAQLGTGGSSGYQYLRSTLSDRYKVFLDLFNLSTFLIPRGYIPPLTRHMKSKLSISRDLIRN---WNDATDSD
N. lecontei          (318) MVQRMIGSQQLGTGGSSGYQYLRSTLSDRYKVFLDLFNLSTFLIPRNLIPPLSRDMKAKLSGAWGCWGA----------D

                           401                      425
H. zea               (393) NAQNGDNAQNGIESSL---------
S. litura            (394) HSQNSQKETNGEGPETTLWQ-----
B. mori              (390) ETTPQNGLEASL-------------
H. virescens         (393) NEQNGIESSF---------------
P. interpunctella    (388) ETHDGELNNCMLKLELA--------
P. polytes           (393) NSQNGLEFL----------------
B. anynana           (396) NKKNGENHQNGLDEFSKLSLNEKKS
A. aegypti           (385) AKQINYAAK----------------
A. darlingi          (386) ATRNGTLH-----------------
A. gambiae           (386) ARNGSLH------------------
Z. navadensis        (395) EVDKPLERSVEASI-----------
N. lecontei          (388) DDQNNLESSAEASM-----------
```
