# Supplementary material for: CRISPR/Cas9 mediated high efficiency knockout of the eye color gene Vermillion in Helicoverpa zea (Boddie)
Source: PLoS One. 2018 May 17;13(5):e0197567. doi: 10.1371/journal.pone.0197567 (PMC5957398; doi:10.1371/journal.pone.0197567)
Supplement: S2 Fig — A maximum likelihood evolutionary tree was constructed using TO sequences from H. zea (MG976796), Heliothis virescens (PCG74852.1), Spodoptera litura (XP_022823393.1), Plodia interpunctella (AAR24625.1), Bicyclus anynana (XP_023952864.1), Papilio polytes (XP_013141355.1), Bombyx mori (XP_004922659.1), Anopheles darlingi (ETN63322.1), Anopheles gambiae (XP_312204.2), Aedes aegypti (AAL37360.1), Neodiprion lecontei (XP_015514842.1), and Zootermopsis nevadensis (XP_021924784.1). Using the MEGA 6.0 we identified the Jones-Taylor-Thornton model with a discrete gamma distribution and some evolutionarily invariable sites (JTT+G+I) as the best model for gene phylogeny reconstruction. JTT+G+I model was run with 10,000 bootstrap replicates was used to generate the evolutionary tree. Lepidopteran and dipteran TO sequences were grouped in two distinct evolutionary branches with 100% bootstrap support. Hymenoptera (N. lecontei) and isopteran (Z. nevadensis) TO sequences were basal to the evolutionary tree with weak bootstrap support. (PDF) [file pone.0197567.s002.pdf]

S2 Fig. The evolutionary tree generated using tryptophan 2,3-dioxygenase (TO) polypeptide sequences from various insect species. A maximum likelihood evolutionary tree was constructed using TO sequences from *H. zea* (MG976796), *Heliothis virescens* (PCG74852.1), *Spodoptera litura* (XP_022823393.1), *Plodia interpunctella* (AAR24625.1), *Bicyclus anynana* (XP_023952864.1), *Papilio polytes* (XP_013141355.1), *Bombyx mori* (XP_004922659.1), *Anopheles darlingi* (ETN63322.1), *Anopheles gambiae* (XP_312204.2), *Aedes aegypti* (AAL37360.1), *Neodiprion lecontei* (XP_015514842.1), *and Zootermopsis nevadensis* (XP_021924784.1). Using MEGA 6.0 [1] we identified the Jones-Taylor-Thornton model [2] with a discrete gamma distribution and some evolutionarily invariable sites (JTT+G+I) as the best model for evolutionary tree reconstruction. Consensus tree constructed by running JTT+G+I model with 10,000 bootstrap replicates [3] was used to represent evolutionary history of the TO gene of the taxa analyzed. Proportion of bootstrap support for nodes are shown above each node. Lepidopteran (red subtree) and dipteran (blue subtree) TO sequences were grouped in two distinct evolutionary branches with 100% bootstrap support. Bootstrap support for grouping *H. zea* with *H. virescens* was 50% and *S. litura* was grouped with *H. zea/H/ virescens* branch in 69% of the bootstrap replicates. Hymenopteran (*N. lecontei*) and isopteran (*Z. nevadensis*) TO sequences were basal to the evolutionary tree with weak bootstrap support.

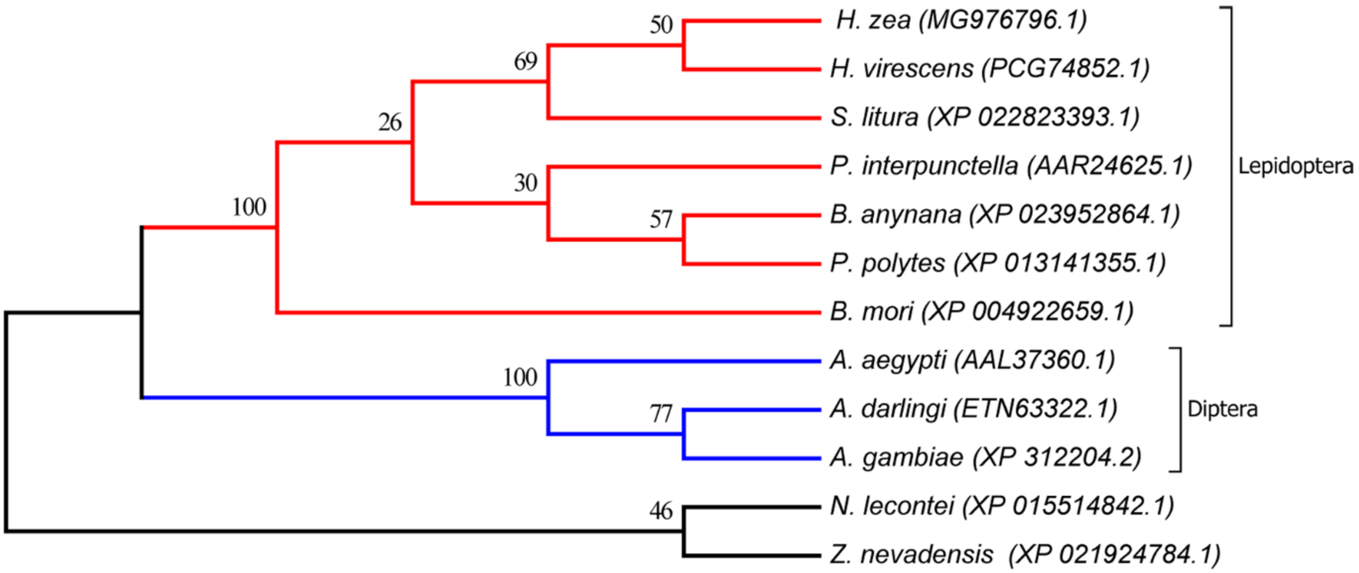

References

1. Jones D.T., Taylor W.R., and Thornton J.M. (**1992**). The rapid generation of mutation data matrices from protein sequences. *Computer Applications in the Biosciences* **8**: 275-282.

2. Tamura K., Stecher G., Peterson D., Filipski A., and Kumar S. (**2013**). MEGA6: Molecular Evolutionary Genetics Analysis version 6.0. *Molecular Biology and Evolution*30: 2725-2729.

3. Felsenstein J. (**1985**). Confidence limits on phylogenies: An approach using the bootstrap. *Evolution* **39**:783-791.
