# Supplementary material for: CRISPR/Cas9 mediated high efficiency knockout of the eye color gene Vermillion in Helicoverpa zea (Boddie)
Source: PLoS One. 2018 May 17;13(5):e0197567. doi: 10.1371/journal.pone.0197567 (PMC5957398; doi:10.1371/journal.pone.0197567)
Supplement: S3 Fig — Nucleotide sequences of the crRNA used in the experiments are shown in bold red text followed by protospacer adjacent sequence (PAM) in bold black text. (PDF) [file pone.0197567.s003.pdf]

S3 Fig. CRISPR RNA (crRNA) designs for *Helicoverpa zea* tryptophan 2,3-dioxygenase (TO) gene exon 6 generated by tools at crispr.mit.edu. Nucleotide sequences of the crRNA used in the experiments are shown in bold red text followed by protospacer adjacent sequence (PAM) in bold black text.

```
LOCUS       Hz_TO_Ex6                     145 bp    DNA
DEFINITION  guides analysis for job Hz_TO, by op.perera@ars.usda.gov on the web at crispr.mit.edu
ACCESSION   5955886094736145
FEATURES             Location/Qualifiers
     protein_bind    complement(14..36)
                     /bound_moiety="CRISPR reverse-strand guide #28940829
                     TCGATCAGAGCTAGTAGAGCTGG"
                     /note="{"n_genic_offtargets": 2, "n_offtargets": 2,
                     "score": "100%", "sequence": "TCGATCAGAGCTAGTAGAGCTGG"}"
     protein_bind    complement(56..78)
                     /bound_moiety="CRISPR reverse-strand guide #28940828
                     AACCCGTGAGTCGTGAGGCCGGG"
                     /note="{"n_genic_offtargets": 2, "n_offtargets": 3,
                     "score": "100%", "sequence": "AACCCGTGAGTCGTGAGGCCGGG"}"
     protein_bind    complement(57..79)
                     /bound_moiety="CRISPR reverse-strand guide #28940827
                     GAACCCGTGAGTCGTGAGGCCGG"
                     /note="{"n_genic_offtargets": 1, "n_offtargets": 1,
                     "score": "100%", "sequence": "GAACCCGTGAGTCGTGAGGCCGG"}"
     protein_bind    complement(61..83)
                     /bound_moiety="CRISPR reverse-strand guide #28940826
                     AGTTGAACCCGTGAGTCGTGAGG"
                     /note="{"n_genic_offtargets": 1, "n_offtargets": 3,
                     "score": "100%", "sequence": "AGTTGAACCCGTGAGTCGTGAGG"}"
     protein_bind    complement(97..119)
                     /bound_moiety="CRISPR reverse-strand guide #28940825
                     TGAGTTTGTTGACAGCAGCCTGG"
                     /note="{"n_genic_offtargets": 3, "n_offtargets": 4,
                     "score": "99%", "sequence": "TGAGTTTGTTGACAGCAGCCTGG"}"
     protein_bind    18..40
                     /bound_moiety="CRISPR forward-strand guide #28940814
                     CTCTACTAGCTCTGATCGAGCGG"
                     /note="{"n_genic_offtargets": 1, "n_offtargets": 2,
                     "score": "100%", "sequence": "CTCTACTAGCTCTGATCGAGCGG"}"
     protein_bind    21..43
                     /bound_moiety="CRISPR forward-strand guide #28940815
                     TACTAGCTCTGATCGAGCGGTGG"
                     /note="{"n_genic_offtargets": 0, "n_offtargets": 1,
                     "score": "100%", "sequence": "TACTAGCTCTGATCGAGCGGTGG"}"
     protein_bind    25..47
                     /bound_moiety="CRISPR forward-strand guide #28940816
                     AGCTCTGATCGAGCGGTGGTTGG"
                     /note="{"n_genic_offtargets": 1, "n_offtargets": 1,
                     "score": "100%", "sequence": "AGCTCTGATCGAGCGGTGGTTGG"}"
     protein_bind    54..76
                     /bound_moiety="CRISPR forward-strand guide #28940819
                     CACCCGGCCTCACGACTCACGGG"
                     /note="{"n_genic_offtargets": 0, "n_offtargets": 1,
                     "score": "100%", "sequence": "CACCCGGCCTCACGACTCACGGG"}"
     protein_bind    67..89
                     /bound_moiety="CRISPR forward-strand guide #28940821
                     GACTCACGGGTTCAACTTCTGGG"
                     /note="{"n_genic_offtargets": 1, "n_offtargets": 2,
                     "score": "100%", "sequence": "GACTCACGGGTTCAACTTCTGGG"}"
     protein_bind    68..90
                     /bound_moiety="CRISPR forward-strand guide #28940822
                     ACTCACGGGTTCAACTTCTGGGG"
                     /note="{"n_genic_offtargets": 5, "n_offtargets": 5,
                     "score": "99%", "sequence": "ACTCACGGGTTCAACTTCTGGGG"}"
     protein_bind    79..101
                     /bound_moiety="CRISPR forward-strand guide #28940823
                     CAACTTCTGGGGCAAGTTCCAGG"
                     /note="{"n_genic_offtargets": 4, "n_offtargets": 9,
                     "score": "99%", "sequence": "CAACTTCTGGGGCAAGTTCCAGG"}"
     protein_bind    103..125
                     /bound_moiety="CRISPR forward-strand guide #28940824
                     TGCTGTCAACAAACTCATTAAGG"
                     /note="{"n_genic_offtargets": 7, "n_offtargets": 14,
                     "score": "98%", "sequence": "TGCTGTCAACAAACTCATTAAGG"}"
ORIGIN
        1 atcagaacaa gagccagctc tactagctct gatcgagcgg tggttggagc gtacacccgg cctcacgact cacgggttca
       81 acttctgggg caagttccag gctgctgtca acaaactcat taaggatgat attgatgctg cttct
```
