# Supplementary material for: CRISPR/Cas9 mediated high efficiency knockout of the eye color gene Vermillion in Helicoverpa zea (Boddie)
Source: PLoS One. 2018 May 17;13(5):e0197567. doi: 10.1371/journal.pone.0197567 (PMC5957398; doi:10.1371/journal.pone.0197567)
Supplement: S4 Fig — Nucleotides identical to the exon 6 of the reference sequence (accession: MF598173) are indicated by a dot (.) and alignment gaps are shown by a hyphen (-). Nucleotide that differ from the reference are indicated on each sequence. CRISPR RNA (crRNA) target sequences and protospacer adjascent motifs (PAM) are underlined with dotted lines and solid lines, respectively. (PDF) [file pone.0197567.s004.pdf]

S4 Fig. Alignment of the partial nucleotide sequences of the exon 6 of the TO gene from reference sequence (Ref), uninjected wild type (WT), eye color mutants from CRISPR/Cas9 injections (IM). Nucleotides identical to the exon 6 of the reference sequence (accession: MF598173) are indicated by a dot (.) and alignment gaps are shown by a hyphen (-). Nucleotide that differ from the reference are indicated on each sequence. CRISPR RNA (crRNA) target sequences and protospacer adjascent motifs (PAM) are underlined with dotted lines and solid lines, respectively.

```
Ref   ATCAGAACAAGAGCCAGCTCTACTAGCTCTGATC---------------GAGCGGTGGTTGGAGCGTACACCCGGCCTCAC---------------------------------GACTCACGGGTTCAACT-TCT-GGGGCAAGTT
WT    ..................................---------------................................---------------------------------.................-...-..........
IM 25 ..................................-------------------......................A.....----------------------------------------..........-...-..........
IM 26 .............................--------------------------------------------------..----------------------------------------..........-...-..........
IM 27 ..................................T--------------................................----------------------------------------..........-...-..........
IM 28 ..................................T--------------......G.........................----------------------------------------..........-...-..........
IM 29 .................................T---------------..AG......A.........AC.....G..C.----------------------------------------.T..
IM 30 ..................................---------------.G.G......G.....................----------------------------------------.......T..-...-..........
IM 31 ..................................---------------...G............................----------------------------------------.......T..-...-..........
IM 32 ..................................---------------......G............GGG..........GCTGCAGTCCAC---G------------------....CA....GG..GA-.A.-..........
IM 33 ..................................---------------...G............................----------------------------------------..........-..G-..........
IM 34 .................................GGAGT------------A..............................----------------------------------------T.........-...-..........
IM 35 ................................--------------------------------------------A...G----------------------------------------..........-...-..........
IM 36 ................................A.G--------------...G.TC...A.CG...G.TG...C.....C.CCGCCGCACCGAC------------------TTCAGTCAC........G-...-..........
IM 37 .................................T---------------GCCCT...A..C......T.........G...----------------------------------------A........G-...-..........
IM 38 .................................TG--------------...CT.G..........A..............----------------------------------------..A.......-...-..........
IM 39 .................................------------------------------------------AG.GGG----------------------------------------...G...GA.-...-..........
IM 40 .................................------------------------------------------A.....----------------------------------------...A......-...-..........
IM 41 .................................T--------------------------------------------..G----------------------------------------..........-...-..........
IM 42 ..................................-------------------...........G................------------------------------------TCAC..........-...-..........
IM 43 .................................------------------------------------------A.....----------------------------------------..........-...-..........
IM 44 ................................-------------------..............................GGGTT----------------------------CACTCAC..........-...-..........
IM 45 ..................................---------------...G............................----------------------------------------..........-...-..........
IM 46 .................................----------------------------------------T.A.....----------------------------------------..........-...-..........
IM 47 .................................------------------------------------------A.....----------------------------------------..........-...-..........
IM 48 .................................ACTAGGTTGGAGCTCAGACC..G...C...............G.....------------------------------------TCACC...G..G..-..C-..........
IM 49 ................................----------------------------------------------...----------------------------------------..........-...-..........
IM 50 .................................T-------------------..A..........C.G......G...G.----------------------------------------A..G......-..G-..........
IM 51 ...G.GC...ACTTG..G.G.----T...G....---------------.G..A.A..GCT.GTTC.G.CAGA..TT....CCGT--------------------------------TTAT.TAG.T..T.AC..
IM 52 T.A.A..A....C.GGC.......T...AA....T--------------...G..G..GG....G.AC.C...CC..C.C.C------------------------------------C--CCCCA.GT..TA.A
IM 53 ..A.T..A.....AACC....T.CCC....T..AAC-------------...T..GT.GG..G...AC....--..C.C.----------------------------------------.CC..CC.GA.GC
IM 54 ..-.A..A..A.A.....C..TAC.A...CA.A.---------------..TG......A..G.GAA.G.....C.AC.C.------------------------------------------CCC.C...-T
IM 55 ..GC...GGG.GA.GCC....C.A.TA...C..AAC-------------...G..G..GG..G...A..C...CC..C.C.TCT-----------------------------------TCC...TTT.GG-..
IM 56  ...................................T--------------...............................---------------------------------------...........-...-..........
IM 57 ..................................-------------------............................----------------------------------------..........-...-..........
IM 58 ................................G.-------------------............................----------------------------------------..........-...-..........
IM 59 .................................----------------..G.......G..GGA.......C.-----------------------------------------------..........-...-.........T.
IM 60 ..................................G--------------......G.........................---------------------------------------...........-...-..........
IM 61 ..................................TAACTGCG-------...A.....GG............GC.G.....----------------------------------------..........-...-..........
IM 62 ................................G.--------------------.C...G......G.....AC.......T--------------------------------------......G....-...-..........
IM 63 .................................----------------...G..G..GG....A.G....G.........ACACACACTCA-------------------------------........-...-..........
IM 64 ..................................-------------------.....G.........GATG.........----------------------------------------..........-...-..........
IM 65 ................................------------CTAAC.....................T..........----------------------------------------..........-...-..........
IM 66 .................................-----------GCTGC...............................TATACACGGACTCATGA-------------------...............-...-..........
IM 67 ................................-----------------.........................------------------------------------------...............-...-..........
IM 68 .................................----------AGCTGAGA..............................TACAA------------------------------...............-...-..........
IM 69 ..................................-----------------GGGGGGGGGGGGGTGG..............GCCCAAA----------------------------...............-...-..........
IM 70 ..................................------------------TGAGGGGGGGTGAGAGCTCACCCACGGGCGCTT------------------------------------..........-...-..........
IM 71 ................................-----------------GAGGGGGGTG.....T...C..G.CT-----------------------------------------...............-...-..........
IM 72 ................................------------------GTAGC....CCTGACTGATGGGTGGATGGAACGTACGCC---------------------------...............-...-..........
IM 24 ..................................-------------------..A...G....T.G.G...A.A..C...CG-----------------------------------CATC.A..TC.G.-..C-..........
IM 74 ................................-----------------------ACAACAACAA---------------------------------------------------...............-...-..........
IM 75 ..................................--------AACGGGCAGAGACGGTGGGG....CGT.....GCCGG-------------------------------------...............-...-..........
IM 76 .................................------------------.-...---------------------....----------------------------------------...G..G.A.-...-..........
IM 77 .................................---------------TCTGAGGGGCGGTG....GGG...G........GTCG------------------------------------..........-...-..........
IM 78 .................................GATGA------------------------------------------------------------------------------...............-...-..........
IM 79 ..................................TCTGCT------------------------------------------------------------------------CT.................-...-..........
IM 80 ..............................------------------------------------------------------------------------------------------.T.........-...-.........A
IM 81 .GCAACTAAAATTATTGCACAATTATGCCTGTGC------------------A..---.ACTT..C.-...G-..TT...G-ATTT------------------------------...............-...-..........
IM 82 ....C............................ATATATATATAT-------------------------------------------------------------------AT.................-...-..........
IM 83 .................................----------------ATTGCTCTTAGGTTCAACGTCCGACGTCAGTTGCAAGCT
IM 84 ................................--------------------......G..T.......GG...A.GTCTCGCGGTTGCTGTCGGCC
IM 85 .................................------------------GA....G.G..T.G.-G..........GGTCACAATTTCTGAGGGTGGACAAGTG
IM 86 ....C............................---------------GGGGGGGTGGAGGGGGCACCCGCCCGCCCCCGGTGCACATCCGGGGGCTGAAGTA
```
