# Supplementary material for: CRISPR/Cas9 mediated high efficiency knockout of the eye color gene Vermillion in Helicoverpa zea (Boddie)
Source: PLoS One. 2018 May 17;13(5):e0197567. doi: 10.1371/journal.pone.0197567 (PMC5957398; doi:10.1371/journal.pone.0197567)
Supplement: S5 Fig — One hundred nucleotides from each scaffold, including 38–40 flanking the off-target sequence obtained from wild type insects (WT) and yellow eye (IM) mutants were aligned with H. zea DNA sequence from corresponding scaffold. Off-target sequence is marked in red text and the PAM sequence is underlined. Nucleotides identical to wild type sequence are indicated by a dot (.) and alignment gaps are indicated by a hyphen (-). Nucleotides that are different from the genomic scaffold are shown on each sequence. (A) Alignment of nucleotide sequences corresponding to the off-target site in the scaffold 185 (S185). Nucleotide sequence of the off-target site from the published genome (S185) was significantly different from those obtained from wild type and yellow eye mutants. (B) Alignment of nucleotide sequences corresponding to the off-target site in the scaffold 1210 (S1210). Two different nucleotide variants, one identical to the genomic scaffold 1210 sequence (S2010a) and the other with a number of polymorphisms (S2010b), were identified in the yellow eye mutants. Alignment of S2010a and S1210b (a) indicates that S2010b variants have additional mutations in the protospacer seed sequence and significantly different from the crRNA design for TO. Alignment of the nucleotide sequences similar to S2010a off-target region from wild type and yellow eye mutants (b) indicates that there were no off-target mutations in the insects with edited TO gene. (C) Alignment of nucleotide sequences corresponding to the off-target site in H. zea genomic scaffold 1430 (S1430). Nucleotide sequences from yellow eye mutants indicate that there were no off-target effects in the edited TO gene. (D) Alignment of nucleotide sequences corresponding to the off-target site in H. zea genomic scaffold 1570 (S1570). Nucleotide sequences from yellow eye mutants indicate that there were no off-target effects in the edited TO gene. (PDF) [file pone.0197567.s005.pdf]

S5 Fig. Alignments of nucleotide sequences of the off-target sites in *H. zea* genomic scaffolds 185, 1210, 1430, and 1570 from yellow eye mutants.

One hundred nucleotides from each scaffold, including 37-39 flanking the off-target sequence obtained from wild type insects (WT) and yellow eye (IM) mutants were aligned with *H. zea* DNA sequence from corresponding scaffold. Off-target sequence is marked in red text and the PAM sequence is underlined. Nucleotides identical to wild type sequence are indicated by a dot (.) and alignment gaps are indicated by a hyphen (-). Nucleotides that are different from the genomic scaffold are shown on each sequence. (A) Alignment of nucleotide sequences corresponding to the off-target site in the scaffold 185 (S185). Nucleotide sequence of the off-target site from the published genome (S185) was significantly different from those obtained from wild type and yellow eye mutants. (B) Alignment of nucleotide sequences corresponding to the off-target site in the scaffold 1210 (S1210). Two different nucleotide variants, one identical to the genomic scaffold 1210 sequence (S2010a) and the other with a number of polymorphisms (S2010b), were identified in the yellow eye mutants. Alignment of S2010a and S1210b (a) indicates that S2010b variants have additional mutations in the protospacer seed sequence and significantly different from the crRNA design for TO. Alignment of the nucleotide sequences similar to S2010a off-target region from wild type and yellow eye mutants (b) indicates that there were no off-target mutations in the insects with edited TO gene. (C) Alignment of nucleotide sequences corresponding to the off-target site in *H. zea* genomic scaffold 1430 (S1430). Nucleotide sequences from yellow eye mutants indicate that there were no off-target effects in the edited TO gene. (D) Alignment of nucleotide sequences corresponding to the off-target site in *H. zea* genomic scaffold 1570 (S1570). Nucleotide sequences from yellow eye mutants indicate that there were no off-target effects in the edited TO gene.

A.

```
S185   CAATT--TGGAAATTAGATTTGCTTAATCAATTTGGAAATCGCTGAGTCGTGAGTCGTGAGGTCGACTACAAATTTTGAATTTTAAAGTCTTTTTTT-AAT
  WT   .....--.........................................AT----------.....................................T...
IM 1   .....--.........................................AT----------............................A........T...
IM 2   .....--.........................................AT----------.....................................T...
IM 3   .....--.........................................AT----------..C..................................T...
IM 4   .....--.........................................AT----------.....................................T...
IM 5   .....--.........................................AT----------.....................................T...
IM 6   .....--.........................................AT----------. ...................................T...
IM 7   .....--.........................................AT----------.....................................T...
IM 8   .....--.........................................AT----------.....................................T...
IM 9   .....--.........................................AT----------.....................................T...
IM 10  .....--.........................................AT----------.....................................T...
IM 11  .....--.........................................AT----------.....................................T...
IM 12  .....--.........................................AT----------.....................................T...
IM 13  .....--.........................................AT----------.....................................T...
IM 14  .....--.........................................AT----------. ...................................T...
IM 15  .....--.........................................AT----------.....................................T...
IM 16  .....--.........................................AT----------.....................................T...
IM 17  .....--.........................................AT----------.....................................T...
IM 18  .....--.........................................AT...............................................T...
IM 19  .....--.........................................AT----------.....................................T...
IM 20  .....--.........................................AT----------.....................................T...
IM 21  .....--.........................................AT----------.....................................T...
IM 22  .....--.........................................AT----------.....................................T...
IM 23  .....GC.........................................AT----------.......C.........C...................T...
IM 24  .....GC.........................................AT----------.......C.........C...................T...
IM 25  .....GC.........................................AT----------.......C.........C...................T...
IM 26  .....GC.........................................AT----------.................C...................T...
IM 27  .....GC.........................................AT----------.......C.........C...................T...
IM 28  .....GC.........................................AT----------.......C.........C...................T...
IM 29  .....GC.........................................AT----------.......C.........C...................T...
IM 30  .....GC.........................................AT----------.................C...................T...
IM 31  .....GC.........................................AT----------.......C.........C...................T...
IM 32  .....AC.........................................AT----------.................C...................T...
IM 33  .....AC.........................................A.----------.................C...................T...
IM 34  .....AC.........................................AT. ..........A....C..C......C...................T...
IM 35  .....AC.........................................AT----------..G..............C...................T...
IM 36  .....AC.........................................AT----------.......C.........C...................T...
IM 37  .....AC.........................................AT----------.................C...................T...
IM 38  .....AC.........................................AT----------.................C...................T...
IM 39  .....AC.........................................AT----------.......C.........C...................T...
IM 40  .....AC.........................................AT----------.................C...................T...
IM 41  .....AC.........................................AT----------.................C...................T...
IM 42  .....AC.........................................AT----------.................C...................T...
IM 43  .....AC.........................................AT----------.................C...................T...
IM 44  .....AC.........................................AT----------.................C...................T...
IM 45  .....AC.........................................AT----------.................C...................T...
IM 46  .....AC.........................................AT----------.................C...................T...
IM 47  .....AC.........................................AT----------.................C...................T...
IM 48  .....AC.........................................AT----------.................C...................T...
```

B.

a)

```
S1210a    GTATTTATTTCAAGTAAAATATTTTCTTGATACAAAA----GCC---------TCACGACTCA--CGTACAAATCCTTCAGATGTGAG--ACGTACAATG
S1210b    A...................G....T...GCT.TTCTTTGT...CCCTATATG..G...TG..TT.A.GTG.TGGA..A.TT...TC.TT.ACG..TG.A

S1210a    GTTTTGT-TCTATTAAAA
S1210b    .C.A.C.C.A.....GGT
```

b)

```
S1210 GTATTTATTTCAAGTAAAATATTTTCTTGATACAAAAGCCTCACGACTCACGTACAAATCCTTCAGATGTGAGACGTACAATGGTTTTGTTCTATTAAAA
  WT  ....................................................................................................
IM 1  ....................................................................................................
IM 3  ....................................................................................................
IM 4  .................................................................A..................................
IM 5  .................................................................A..................................
IM 7  ....................................................................................................
IM 8  ....................................................................................................
IM 10 ....................................................................................................
IM 11 .................................................................A..................................
IM 12 ....................................................................................................
IM 14 ....................................................................................................
IM 15 ....................................................................................................
IM 17 ....................................................................................................
IM 20 ....................................................................................................
IM 21 ....................................................................................................
IM 22 ....................................................................................................
IM 23 ....................................................................................................
IM 24 ....................................................................................................
IM 26 ....................................................................................................
IM 28 ....................................................................................................
IM 30 ....................................................................................................
IM 34 ....................................................................................................
IM 35 ....................................................................................................
IM 37 ....................................................................................................
IM 38 ....................................................................................................
IM 40 ....................................................................................................
IM 41 ....................................................................................................
IM 43 ....................................................................................................
IM 45 ....................................................................................................
IM 47 ....................................................................................................
IM 48 ....................................................................................................
```

C.

```
S1430 TAAAATAGCGACACTCGATTGGCAATGTTGCGAAGGATATGAGATGAACATTCTGATCGAGCGGAAATCCAATAAAAGTATGAGGGAGCGACATCGACTT
  WT  ....................................................................................................
IM 1  ....................................................................................................
IM 2  ....................................................................................................
IM 3  ....................................................................................................
IM 4  ....................................................................................................
IM 5  ....................................................................................................
IM 6  ....................................................................................................
IM 7  ................C...................................................................................
IM 8  ....................................................................................................
IM 9  ....................................................................................................
IM 10 ....................................................................................................
IM 11 ....................................................................................................
IM 12 ....................................................................................................
IM 13 ....................................................................................................
IM 14 .......TG...........................................................................................
IM 15 ....................................................................................................
IM 16 ....................................................................................................
IM 17 ....................................................................................................
IM 18 ....................................................................................................
IM 19 ....................................................................................................
IM 20 ....................................................................................................
IM 21 ....................................................................................................
IM 22 ....................................................................................................
IM 23 ....................................................................................................
IM 24 ................C...................................................................................
IM 25 ....................................................................................................
IM 26 ....................................................................................................
IM 27 ....................................................................................................
IM 28 ....................................................................................................
IM 29 ....................................................................................................
IM 30 ....................................................................................................
IM 31 ....................................................................................................
IM 32 ....................................................................................................
IM 33 ....................................................................................................
IM 34 ....................................................................................................
IM 35 ....................................................................................................
IM 36 .TC.................................................................................................
IM 37 ....................................................................................................
IM 38 ....................................................................................................
IM 39 ....................................................................................................
IM 40 ....................................................................................................
IM 41 ................C...................................................................................
IM 42 ....................................................................................................
IM 43 ....................................................................................................
IM 44 ....................................................................................................
IM 45 ....................................................................................................
IM 46 ....................................................................................................
IM 47 ....................................................................................................
IM 48 ....................................................................................................
```

D.

```
S1570 AAAATAAAAAAGTAATAAAATAAGAGATGTTTACGAGCGTAAAATGACGTGAGTCGTGAGGCCGCGCCACTTGGGCTGTTTACAGCAGTCAGCAGCGCAG
  WT  ....................................................................................................
IM 1  ....................................................................................................
IM 2  ....................................................................................................
IM 3  ....................................................................................................
IM 4  ....................................................................................................
IM 5  ....................................................................................................
IM 6  ....................................................................................................
IM 7  ....................................................................................................
IM 8  ....................................................................................................
IM 9  ..G.................................................................................................
IM 10 ....................................................................................................
IM 11 ....................................................................................................
IM 12 ....................................................................................................
IM 13 …................................................................................................
IM 14 ....................................................................................................
IM 15 ....................................................................................................
IM 16 ....................................................................................................
IM 17 ....................................................................................................
IM 18 ..G.................................................................................................
IM 19 ....................................................................................................
IM 20 ....................................................................................................
IM 21 ....................................................................................................
IM 22 ....................................................................................................
IM 23 ....................................................................................................
IM 24 ....................................................................................................
IM 25 ....................................................................................................
IM 26 ....................................................................................................
IM 27 ....................................................................................................
IM 28 ..G.................................................................................................
IM 29 ....................................................................................................
IM 30 ....................................................................................................
IM 31 ....................................................................................................
IM 32 ....................................................................................................
IM 33 ....................................................................................................
IM 34 ....................................................................................................
IM 35 ....................................................................................................
IM 36 ..G.................................................................................................
IM 37 ....................................................................................................
IM 38 ....................................................................................................
IM 39 ....................................................................................................
IM 40 ....................................................................................................
IM 41 ....................................................................................................
IM 42 ....................................................................................................
IM 43 ....................................................................................................
IM 44 ....................................................................................................
IM 45 ....................................................................................................
IM 46 ....................................................................................................
IM 47 ....................................................................................................
IM 48 ....................................................................................................
```
