# Supplementary material for: CRISPR/Cas9 mediated high efficiency knockout of the eye color gene Vermillion in Helicoverpa zea (Boddie)
Source: PLoS One. 2018 May 17;13(5):e0197567. doi: 10.1371/journal.pone.0197567 (PMC5957398; doi:10.1371/journal.pone.0197567)
Supplement: S1 Table — Seed sequence (12 nucleotides upstream of the protospacer adjacent motif) of the target sequences of the TO gene was used to search genome of H. zea to identify potential off-targets that had three or less mismatches with the seed sequences of the targets and ten or less total mismatches. Primer pairs designed to PCR amplify and sequence off-target sequences are shown next to the selected sequences. Seed sequence region is shown in blue text and nucleotide mismatches are shown in lowercase text. PAM sequences are shown in bold black text. A lower case “c” at the end of the start position indicates an off-target sequence on the complementary strand of a scaffold. N/A: primers were not designed to these off-targets. (PDF) [file pone.0197567.s006.pdf]

S1 Table. Potential off-targets in the genome of *Helicoverpa zea*. Seed sequences (12 nucleotides upstream of the protospacer adjacent motif) of the target site 1 (**GCTCTGATCGAG**) and target site 2 (**CCGTGAGTCGTG)** of the TO gene were used to search genome of *H. zea* to identify potential off-targets that had three or less mismatches with the seed sequences of the targets and ten or less total mismatches. Primer pairs designed to PCR amplify and sequence off-target sequences are shown next to the selected sequences. Seed sequence region is shown in blue text and nucleotides not matching the target sequences are shown in lowercase text. PAM sequences are shown in bold black text. A lower case "c" at the end of the start position indicates an off-target sequence on the complementary strand of a scaffold. N/A: primers were not designed to these off-targets.

gRNA target site	Genomic Scaffold #	Off-target start position	Off target sequence	PAM	Total mismatch #	Forward PCR Primer (5' to 3')	Reverse PCR primer (5' to 3')	Amplicon size (bp)
1	1430	28347	AgaTgaac**atTCTGATCGAG**	CGG	8	AAACCACACTTAGTTGAGAGGCA	TTGCCCAGTTAAATCTTCGA	413
2	185	258131	TcgctgAgt**CGTGAGTCGTG**	AGG	8	AAATTAAGTGATGCTGCCGTAAG	TGGTGGAAACGGTACATTTAGC	567
2	1210	80173	GGaTttgt**aCGTGAGTCGTG**	AGG	7	GTGAATGTATCTGGGACGGTTC	GATGACGACGGACGAGGTCT	1172
2	1570	50187	GtaaaAtg**aCGTGAGTCGTG**	AGG	7	TGAGTAATAAACAAGGAAACCGC	ATTGGTTTAGCTTTCGGCGT	416
1	522	165033	AgtTAgcA**GCTCTGcCtGAG**	TGG	6	N/A	N/A	N/A
1	1430	18518	TTCTAtct**GtTCTGcaCGAG**	TGG	7	N/A	N/A	N/A
2	40	528393c	TGTgaAAC**CCGTGAcTgGTa**	CGG	6	N/A	N/A	N/A
2	40	548156c	AtacattC**CCGTGAcTCaTa**	TGG	9	N/A	N/A	N/A
2	180	97132c	CaTcccAg**gaGTGAGTCGTG**	AGG	8	N/A	N/A	N/A
2	185	19054	TaTacAtt**CtGTGAGTCcaG**	CGG	9	N/A	N/A	N/A
2	2020	29321	AGgaaAta**aaGTGAGTCGTG**	AGG	7	N/A	N/A	N/A
2	2020	34742c	cccTcgct**CCtgGgGTCGTG**	CGG	9	N/A	N/A	N/A
